# Identification and management of infections caused by *Giardia* sp., *Trichuris* sp. and *Demodex* sp. in captive Brazilian porcupines (*Coendou prehensilis*)

**DOI:** 10.1016/j.ijppaw.2024.100976

**Published:** 2024-08-30

**Authors:** Gastón Moré, Peggy Rüegg-van den Broek, Olivier J. Glardon, Diana S. Gliga, Caroline F. Frey, Walter Basso

**Affiliations:** a Institute of Parasitology, University of Bern, Länggassstrasse 122, 3012, Bern, Switzerland; b National Scientific and Technical Research Council (CONICET), Godoy Cruz 2290, C1425FQB, Buenos Aires, Argentina; c Papiliorama Foundation, 3210, Kerzers, Switzerland

**Keywords:** *Trichuri*s sp, *Giardia* sp., *Demodex* sp., Treatment, Colony management

## Abstract

The Brazilian porcupine (*Coendou prehensilis*, Rodentia, Erethizontidae) is an arboreal South American nocturnal rodent. Switzerland is home to one of the largest captive colonies in Europe. In June 2022, most of the animals in this colony showed severe diarrhoea, and *Giardia* sp. cysts were detected. All the animals were treated with metronidazole (75 mg/animal/day orally) for five days, repeating after two weeks. The diarrhoea continued, sometimes containing blood, and further analyses revealed *Giardia* sp. cysts and *Trichuris* sp. eggs with a particular undulating eggshell in pooled samples. The soil layer of some enclosures was removed to thoroughly clean and disinfect the underlying concrete floor. The animals were treated with fenbendazole (50 mg/kg/day orally) for 5 days repeating after three days. *Giardia* sp. cysts were not further detected. However, *Trichuris* sp. eggs were detected in branch bark samples and in six animals 2–3 months after treatment. The treatment with fenbendazole was repeated and no further *Trichuris* sp. eggs were detected. A *18S rRNA* fragment consensus sequence showed 98.58% identity with *Trichuris fossor*. The *Trichuris* sp. in *C. prehensilis* may represent a new species, specific for arboreal porcupines. *Demodex* mites were observed in faecal flotations and thereafter in skin scrapings from five animals (four of them being family-related). A *16S* consensus sequence showed 86.4% identity with other *Demodex* species. The animals were initially treated with moxidectin (0.4 and 0.8 mg/kg orally) and afterwards with sarolaner (10 mg/animal) but the treatments were not completely effective since in control scrapings, two animals evidenced few non-motile mites. An individual susceptibility and poor immunological control of the infection is suggested. Treatment with fenbendazole was effective against *Giardia* sp. and *Trichuris* sp. infections; however, reinfections may occur if the enclosures and tree branches are not deep cleaned and disinfected or replaced.

## Highlights

• *Trichuris* sp. and *Giardia* sp. infections were associated with diarrhoea in *Coendou prehensilis*.
• Treatment with febendazole was effective against *Giardia* sp. and *Trichuris* sp. infections.
• *Demodex* sp. mites were detected in five animals, four being family related.
• Monthly treatment with moxidectin and sarolaner were partially effective against *Demodex*.
• Arboreal porcupines may harbour host-specific *Trichuris* and *Demodex* species.

## Graphical abstract

**Figure:**
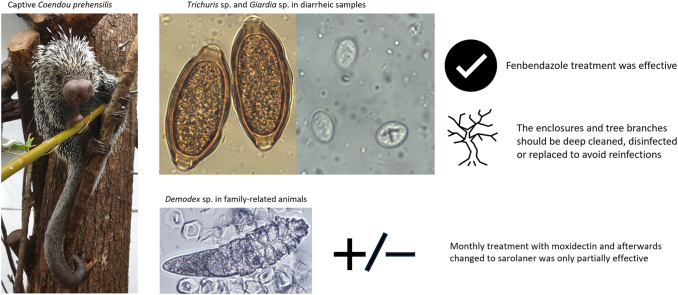

## 1 Introduction

The Brazilian porcupine (*Coendou prehensilis*, Rodentia, Erethizontidae) is a nocturnal arboreal rodent, natural resident of central and northern regions of South America. These animals, also named prehensile-tailed porcupines, are herbivores that forage mostly among trees. They eat the bark and the cambium layer of some trees, as well as buds, fruits, roots, stems, blossoms, leaves, un-ripened seeds, and if in proximity, crops like bananas and corn. According to the International Union for Conservation of Nature (IUCN) *C. prehensilis* is listed as Least Concern (Marinho-Filho and Emmons, 2016). However, increasing human encroachment may reduce the habitat of porcupines, which could ultimately lead to a decline in their population.

In Europe, there are around 50 zoo-kept specimens registered at the European Association of Zoos and Aquaria, and one of the biggest colonies (around 14 animals with active reproduction, divided in groups) is at Papiliorama, Switzerland, a zoological garden harbouring tropical plants and animals, as well as native wildlife and a petting farm (https://www.papiliorama.ch/en/).

Little is known about parasitosis in coendous. It has been reported that *Blastocystis* sp. infection was associated with diarrhoea in a young animal (Goe et al., 2016). In 45 rescued free-ranging specimens in French Guiana several haemoparasites were detected: microfilariae (n = 23), *Babesia* spp. (n = 3), Trypanosomatidae (n = 3), and *Hepatozoon* sp. (n = 1) (de Thoisy et al., 2000). There is one report in which the porcupines were used as sentinels, evidencing severe clinical signs after natural infection with *Leishmania chagasi* (Le Pont et al., 1989). In a road-killed porcupine in Brazil, *Amblyomma longirostre* harbouring a new *Rickettsia* sp. was described (Labruna et al., 2004). Severe sarcoptic mange has been identified in another arboreal porcupine, the *Coendou quichua* in Colombia (Gonzalez-Astudillo et al., 2018).

There are no published studies of diagnosis, clinical implications, and treatment of gastrointestinal or cutaneous parasitic infections in *C. prehensilis*. Other porcupine species could be infected with at least four different *Trichuris* spp. (Cavallero et al., 2021; Rivero et al., 2022). The *Trichuris* sp. from the crested porcupine (*Hystrix cristata*) was phylogenetically identified as sister species from those affecting other rodents (Rivero et al., 2022). In addition, South and North American rodents also harbour a high spectrum of *Trichuris* spp., with around 28 described species being most of them host species specific, at least at family level (Callejón et al., 2016; Robles et al., 2018). Fenbendazole was the most effective drug at clearing infection with *Trichuris* sp. in a colony of African Pouched Rats (*Cricetomys ansorgei*) (Cullin et al., 2017).

Similarly, *Demodex* spp. have been described affecting several rodent species, most of them being specific for host species or related species (Bukva, 1995; Brosseau, 2020). However, there are no descriptions of *Demodex* sp. infection in porcupines. Recently, oral fluralaner and topical sarolaner treatments have been proven to be effective in treating clinical demodicosis in different rodents (Beck et al., 2021; Brosseau, 2020).

Rodents could also be infected with *Giardia* spp., some of them shared with other mammals and humans, being therefore considered as reservoirs for zoonotic infections in synanthropic environments (Li et al., 2023). The treatment of laboratory Swiss mice with a combination of metronidazole and fenbendazole has been proven to be effective and safe (Bezagio et al., 2017).

The present study aimed to report the diagnosis and management of clinical infections caused by *Giardia* sp., *Trichuris* sp. and *Demodex* sp. in Brazilian porcupines in a captive breeding colony in Switzerland.

## 2 Materials and methods

### 2.1 Study location and sample population

This study was conducted as part of the routine health controls of the *C*. *prehensilis* colony (n = 14 animals; 8 males and 6 females) from Papiliorama, Kerzers, Switzerland (https://www.papiliorama.ch/en/) in collaboration with the Institute of Parasitology, University of Bern, Switzerland, from 2022 to the beginning of 2024. The animals were kept in five defined groups (2–4 animals each) in 5–6 different enclosures. The enclosures were enriched with natural and built branches and the concrete floor was covered with wood chips and earth. The animals were fed 3 times a day: the first feeding in the morning consisted in pellets, the second and third feedings were in the afternoon, consisting mainly of vegetables and fruits. The food was presented in several small metal bowls (at least one per animal) that were placed in a metal ring attached on the trees/branches at least 1.20 m above the ground.

### 2.2 Microscopic parasitological diagnosis

Faecal samples were collected, submitted to the Institute of Parasitology of the Vetsuisse Faculty, University of Bern (IPB), and processed within 24 h of reception. Coproparasitological examination from individual or pooled faecal samples from each group was initially performed by a combined sedimentation-flotation with 44% zinc chloride solution (density 1.320) and by sodium acetate-acetic acid-formalin-concentration (SAFC) methods. After the initial detection of *Trichuris* sp. eggs, faecal samples were re-examined using a more concentrated flotation solution, 66% zinc chloride (density 1.450), resulting in detection of a higher number of eggs on each slide; therefore, this solution was further used to screen for *Trichuris* sp. eggs in all samples.

Additionally, branch bark samples from the enclosures, as well as cockroaches captured with a vacuum machine were collected, ground, and processed by the sedimentation-flotation and SAFC techniques.

In faecal flotations performed in March 2023, *Demodex* sp. mites were observed in samples from three animals (two of them, mother and son, showed skin lesions) and in one pooled sample. After this finding, skin scrapings were performed under anaesthesia in all animals in which *Demodex* sp. was observed in faecal material, as well as in their family-relatives and animals in close contact with them.

### 2.3 Molecular analysis

DNA was extracted from (i) faecal sediments containing *Trichuris* sp. eggs from four samples (three pooled samples from 3 or 4 animals, and one sample from a single animal; Table 1), (ii) concentrated *Trichuris* sp. eggs (n = 100 or 200) from two faecal samples (Table 1), and (iii) *Demodex* sp. mites obtained by skin scraping, using the Quick-DNA™ Fecal/Soil Microbe MiniPrep Kit (Zymo Research, USA), according to manufacturer's instructions. Subsequently, in order to increase the amount and purity of *Trichuris* DNA, a pre-step was applied to the DNA extraction on larvated eggs. Briefly, 100 and 200 eggs that had been stored for 1 year at 4–8 °C, were transferred to two 50 ml tubes each filled with 2% H_2_SO_4_ and left for larvation at room temperature for 2 months. The larvated eggs were transferred to microtubes and washed with sterile water, centrifuged for 5 min at 600×*g*, and submitted to cycles (n = 5) of freezing at −20 °C for 10 min and heating at 95 °C for 10 min. After adding 750 μl of Bashing-beads buffer (from the Quick-DNA™ Fecal/Soil Microbe MiniPrep Kit) and 20 μl of proteinase K (QIAGEN) the tubes were incubated in a thermomixer (F1.5, Eppendorf) at 1000 RPM overnight at 56 °C. After this digestion step, the beads were added, and the sample was processed according to the manufacturer's instructions from the kit (Zymo Research, USA).

**Table 1:** Results of the different PCRs and sequencing performed to identify the *Trichuris* sp. affecting *Coendou prehensilis*.

	Results from each PCR target/sequence identity			
Sample ID-Type	*Cob*	*cox1*	*ITS2*	*18S rRNA*
22D3640-PFS	- (banding)	- (banding)	- (banding)	**+** /656bp^1^* 98.48% identity (90% coverage) with *Trichuris fossor* sequences (MT071353 and MT071354) and 94.39% (100% coverage) with a *Trichuris muris* sequence (HF586907)
22D3662-PFS	- (banding)	**+** /sequencing failed	- (banding)	+/535bp 100% (identity and coverage) with *Caenorhabditis* spp. (MN519140 and others)
23D1326-PFS	**+** /180bp 98.89% with *Eucoleus boehmi* sequences (KX027311 and KR186213)	**+** /sequencing failed	- (banding)	+/390bp 99.5% identity (100% coverage) with *Demodex* spp*.* (KY922187 and others)
23D1327-SFS	- (banding)	- (banding)	- (banding)	–
22D3640-100 E	–	–	–	–
22D3640-200 E	–	–	–	–
22D3662-100 E	–	–	–	–
22D3662-200 E	–	–	–	–
22D3662-100 LE	–	–	–	**-**
22D3662-200 LE	–	–	–	**+** 679bp^2^*, 98.58% identity (90% coverage) with *Trichuris fossor* sequences (MT071353 and MT071354), and 94.58% (100% coverage) with a *Trichuris muris* sequence (HF586907)

Ref.: PFS = pooled fecal sediment; SFS = single animal fecal sediment; E = *Trichuris* sp. eggs; LE = larvated *Trichuris* sp. eggs.^1^ Only forward primer sequence (reverse primer sequence failed).^2^ Consensus sequence, primers trimmed, reported in the Genbank (accession number PP977476). *Both sequences are identical.

*Trichuris* spp. mitochondrial *cob* and *cox1* gene fragments were PCR-amplified with primers D769-D770, and HC02198F-CORA, proceeding essentially as described by Callejón et al. (2016). Additionally, the Internal Transcribed Spacer 2 (*ITS2*) was amplified using primers 5.8SF and ITS2R, proceeding according to the protocol described by Robles et al. (2014), and a fragment of the *18S rRNA* gen using primers 18S 965 and 18S 1573R with the protocol described by Guardone et al. (2013). All PCRs were conducted using *Trichuris suis* DNA (female worm) as positive control and ultrapure water as non-template control.

A fragment of the *Demodex* sp. mitochondrial *16S rDNA* gene was amplified using the primers described by Sastre et al. (2012) (final concentration 2 μM) using the following program: 94 °C/10 min, 40 cycles (94 °C/30 s, 57 °C/30 s, 72 °C/30 s) and final extension of 72 °C/10 min. The DNA of *Demodex caprae* was used as positive control and ultrapure water as non-template control.

All PCRs were conducted in 25 μl final volume, using the 2X Multiplex PCR kit (QIAGEN, Germany) in a GeneAmp PCR System 9700 cycler (Applied Biosystems). The PCR products were separated in an ethidium bromide-stained 1.5% agarose gel by electrophoresis (30 min 120 V) and visualised with a UV light image system (E-Box, Vilber, France).

The PCR products were excised from the gel and purified with the DNA Clean & Concentrator-5 (Zymo Research, USA) according to the manufacturer's instructions and submitted for Sanger sequencing in both directions (using the same primers as for each PCR) to Microsynth, Balgach, Switzerland (https://srvweb.microsynth.ch). Sequences obtained were analysed and aligned using the Geneious Prime software (https://www.geneious.com). Nucleotide BLAST analysis (http://blast.ncbi.nlm.nih.gov/Blast.cgi) was used to compare the obtained consensus sequences with sequences available in the GenBank.

## 3 Results

### 3.1 Diagnosis, treatment and health management carried out

**Fig. 1:**
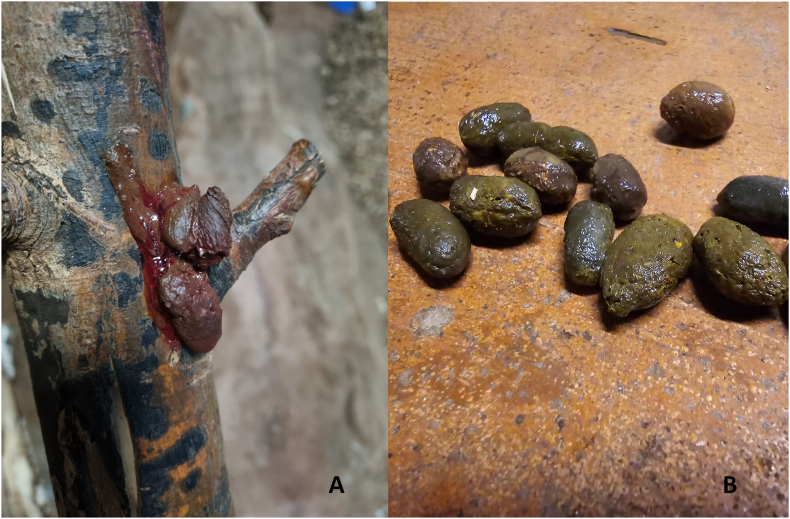
Diarrheic faecal material from a *C. prehensilis*, evidencing undigested blood (A). Normal shaped faecal material of *C. prehensilis* (B).

Between June and August 2022, most of the animals in the colony showed episodes of diarrhoea. Diagnostics revealed *Giardia* sp. cysts by SAFC in four of the groups (pooled samples) and in two individual animals. All the animals were treated with metronidazole (75 mg/animal/day orally) for five days, and repeated after two weeks (September/October 2022). After this treatment the animals received a yogurt-powder supplement orally for three days (Bene-Bac®, PetAg Inc., Hampshire, Illinois, USA). Some animals continued showing diarrhoea, sometimes with blood (Fig. 1A).

**Fig. 2:**
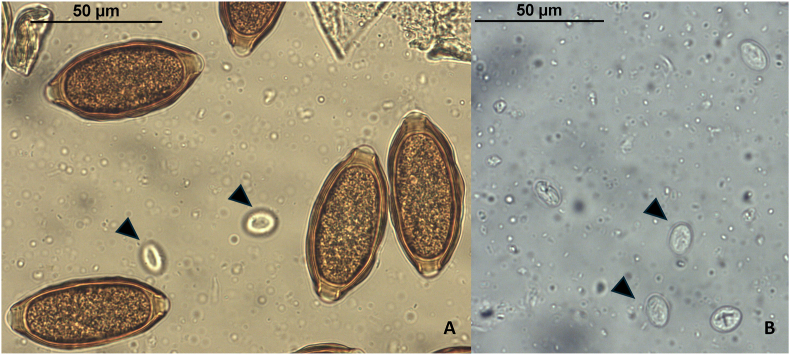
Faecal flotation from a pooled sample showing *Trichuris* sp. eggs (A). The sample contained also *Giardia* sp. cysts (arrowheads), clearly visible in SAFC (B).

Subsequently performed analyses (October to December 2022) revealed moderate to high amounts (≥4 elements per 10X objective field) of *Trichuris* sp. eggs and *Giardia* sp. cysts in pooled samples from three of the groups and in one individual sample of a young animal. The *Trichuris* sp. eggs showed a particular shape, with an undulating surface, and measured on average 66.3 × 28.4 μm (range 58–67.2 μm x 24.5–32.1 μm, n = 25 eggs) (Fig. 2). The undulating surface of the eggs was also observed in native faecal samples and in SAFC, ruling out the possibility of an artifact due to the used flotation solution. Pictures and measurements were conducted with a Nikon Eclipse Ci microscope and a calibrated Nikon Camera (model DFK 23UP031) using the NIS software (Nikon).

The soil layer from four of the enclosures was removed to allow thorough cleaning and disinfection of the concrete floor underneath (brushing with detergent and then with bleach). The animals were treated with fenbendazole (Panacur® 10%) at a dose of 50 mg/kg/day orally for 5 days and repeated after three days (November to December 2022). To provide the medication, the feed pellets were soaked with the total dose for all the animals in each enclosure. No *Giardia* sp. cysts were detected either one week after treatment or during the following twelve months. By the flotation technique performed on pooled samples from each group and on some individual samples in January 2023 (*i.e.*, approx. 1 month after treatment), no *Trichuris* sp. eggs were observed and most of the animals produced normal faeces (0.5–1 cm drops, Fig. 1B). Nevertheless, two to three months after treatment (February to April 2023), *Trichuris* sp. eggs were observed in faeces from one pooled sample (four male animals) and in five individual samples (one of these animals showing bloody diarrhoea). Fenbendazole treatment was repeated, and one week later no *Trichuris* sp. eggs were observed, and the diarrhoea ceased. The bark samples from two enclosures contained a moderate amount of *Trichuris* sp. eggs (morphologically identical to the ones observed in faecal samples). The cockroaches were morphologically identified as *Periplaneta australasie*, and no *Trichuris* sp. eggs or *Giardia* sp. cysts were identified in pooled homogenates of cockroaches. The enclosure of the four *Trichuris*-positive males was cleaned with a pressure washer and disinfected with Neopredisan® 135-1 (4-Chlor-M-Kresol MENNO Chemie-Vertrieb, Norderstedt).

Routine coprological investigations every two-three months until January 2024 did not detect *Giardia* sp. cysts. Only one animal shed *Trichuris* sp. eggs in August 2023. All animals from this enclosure received a further treatment with fenbendazole.

**Fig. 3:**
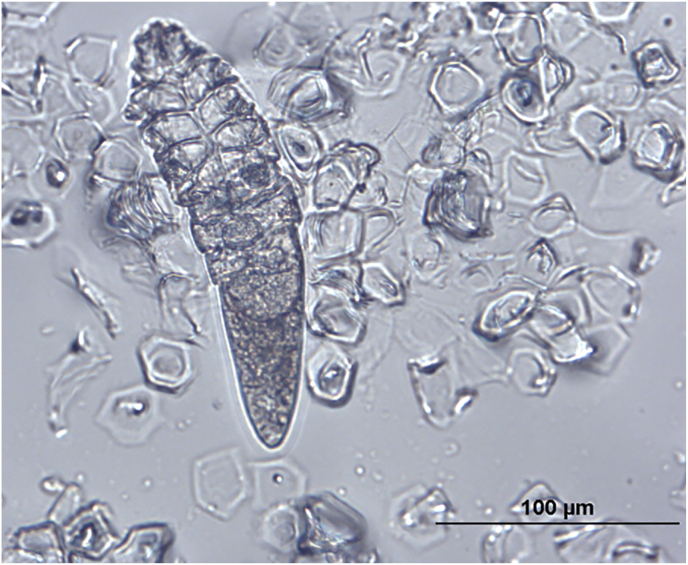
*Demodex* sp. mite detected in skin scraping of a male *C. prehensilis* with skin lesions.

*Demodex* sp. was identified in skin scrapings from five of ten animals, four of which were family-related: a mother and one son, both with dry skin and alopecic regions, and two daughters without evident clinical signs. The observed mites measured on average 206 μm in length and 39 μm in width (range 184–228 x 31.5–45 μm, n = 10) (Fig. 3), the males being shorter (mean 192.4 × 40.3 μm, range 184–198.3 x 34–45 μm, n = 5) than the females (mean 220 × 37.8 μm, range 209.5–228 x 31.5–45 μm, n = 5).

One group of four animals was treated orally with moxidectin (Cydectin® 0.1%; 0.4 mg/kg) in April 2023. One month after treatment, motile *Demodex* sp. adults were still present in the skin scrapings, and it was decided to treat all five positive animals with a double moxidectin dose (Cydectin® 0.1%; 0.8 mg/kg) and to repeat it after one week (May 2023). No adverse effects were recorded in any of the animals. A skin scraping control one month after the second treatment revealed the presence of motile *Demodex* sp. in three animals (all from the same mother), one of them (a male animal) with skin lesions. The treatment was changed to sarolaner (Simparica® 10 mg tablets, Zoetis Schweiz GmbH), one tablet per animal once a month. Oral medication was usually well accepted when offered mixed in cooked whole-wheat pasta or potato or on a spoon with organic peanut butter (without added sugar). After the third sarolaner treatment, two of the animals still evidenced a few non-motile *Demodex* sp. in the scrapings, and the skin improved only slightly. The mother and son with skin lesions treated with sarolaner, showed inflammation of the nose skin one day after each of three treatments. Due to these reactions, the treatment with sarolaner was suspended. Potential bacterial or mycotic infections were not ruled out.

### 3.2 Molecular studies

Table 1 summarizes the PCR results for the four different targets of *Trichuris* spp. The *Trichuris* spp. PCRs resulted in banding patterns in most faecal sediment samples, and the attempts to sequence the selected bands for each PCR failed or produced mixed chromatograms. The samples from concentrated *Trichuris* sp. eggs gave negative results, except for the *18S rRNA* gene-targeted PCR on the sample with 200 larvated eggs (sample 23D3662) (Table 1). The obtained consensus sequence was registered in the GenBank as from *Trichuris* sp. in *C. prehensilis* (accession number PP977476). This sequence of 679 bp (primers trimmed) had an 98.48–99.58% identity (with 90% coverage) with *Trichuris fossor* sequences from rodents of the genus *Thomomys* in USA (MT071353 and MT071354), and 94.39–94.54% (with 100% coverage) with a *Trichuris muris* sequence (HF586907).

The obtained consensus sequence from the *16S rRNA gene*-targeted PCR from mites was 294 bp (primers trimmed) and had a low identity with other reported sequences. The highest matches were *Demodex canis* (86.44%, JF784001) and *D. injai* (86.15%, HE817765), both with 100% coverage. The sequence was submitted to the GenBank as from *Demodex* sp. in *C. prehensilis* (accession number PP982320).

## 4 Discussion and conclusions

This study showed the association of *Giardia* sp. and trichurids infection with diarrhoea episodes in captive Brazilian porcupines. The treatment with fenbendazole (applied with the protocol mentioned here), was effective to stop the cyst and egg shedding. Similarly, fenbendazole treatment was the most effective to clear *Giardia* and *Trichuris* infections in other rodent species (Bezagio et al., 2017; Cullin et al., 2017). Moreover, *Giardia* sp. cysts were not detected during the subsequent coprological analyses, suggesting that the animals were not re-infected. Considering the type of eggs observed and the nature of haemorrhagic diarrhoea episodes (with fresh blood) we considered the trichurids detected as a *Trichuris* sp., probably located in the large intestine (Walden, 1991). However, since no adult specimens could be retrieved so far, our hypothesis could not be confirmed. Given the diversity of *Trichuris* spp. described in rodents, we attempted molecular identification using four different genetic targets (Callejón et al., 2016; Robles et al., 2014; Guardone et al., 2013). Several attempts to amplify specific targets for *Trichuris* spp. rendered inconclusive results. In fact, other agents like *Caenorhabditis* spp. and *Demodex* spp. were also identified with the *18S rRNA* gene-targeted PCR. These results suggest that the primers used are not highly specific and should be applied on pre-selected or purified material (i.e. adult parasites) to render appropriate and specific results. The eggs of *Trichuris* spp. are known to require the use of beads-based kits to be disrupted (Demeler et al., 2013). Additionally, the eggs of this trichurid species are probably highly resistant to lysis, even after applying the mechanical disruption step with beads, as shown by the negative results in the samples that contained only concentrated eggs. The use of larvated eggs, along with the addition of thermal disruption cycles and a pre-digestion step with proteinase K proved partially useful in obtaining amplifiable DNA in the *18S* PCR; however, an inspection of the remaining buffer and beads from the Zymo kit revealed eggs which were not lysed after all treatments (data not shown, pictures available). On the other hand, such lysis “struggle” is to be expected given the long-lasting resistance and survival of the eggs in the environment, which can easily become a source of re-infection. Considering this, the removal of the biological floor of most enclosures was decided, and the concrete floor was disinfected. Despite these measures, the animals considered *Trichuris*-free started to shed eggs 2–3 months later, which is the prepatency period of many trichurids, suggesting reinfection (Walden, 1991). Molecularly, the obtained *18S rRNA* fragment sequence (PP977476) showed only moderate identity with *Trichuris fossor* sequences from rodents of the genus *Thomomys* in USA (MT071353 and MT071354), and with a *Trichuris muris* sequence (HF586907). The distinctive undulating eggshell and the relatively low identity may indicate that the species infecting the presented coendous could represent a novel, previously undescribed species, or a species without a reported *18S rRNA* sequence. However, to confirm that coendous are harbouring some of these species or a new one, the analysis of adult parasites, both morphologically and molecularly, is required (Cavallero et al., 2021; Rivero et al., 2022). We hypothesize that the coendous harbour a specific *Trichuris* sp., different to the ones described in terrestrial porcupines. Moreover, the *Trichuris* sp. in *C. prehensilis* showed a particular undulating eggshell, which differed from the *Trichuris* spp. described in other porcupines and rodents, and could facilitate attachment to the bark surface and hence transmission in arboreal animals (Cavallero et al., 2021; Rivero et al., 2022).

As a first conclusion, the treatment with 50 mg/kg/day fenbendazole orally for 5 days (twice, repeating it after three days) was effective against *Giardia* sp. and *Trichuris* sp. infections in *C. prehensilis*. However, the enclosures including enrichments such as natural and artificial branches should be deep-cleaned and disinfected to avoid re-infections with *Trichuris* sp. Once the infection is detected, the feasibility of replacing branches should be considered.

The *Demodex* sp. mites detected were morphologically alike with species described in rodents, in particular with *D. ratticola* from *Rattus norvegicus* (Bukva 1995). After detecting the mites in some faecal flotations, a protocol for skin scraping sampling under anaesthesia was established. This protocol allowed to identify other animals harbouring mites, some of them evidencing skin lesions. The unexpectedly obtained sequence (with primers intended to amplify *18S rRNA* gene fragments of *Trichuris* spp.) showed a 99.5% identity with 100% coverage with *18S rRNA* sequences from several *Demodex* spp. On the other hand, the sequence obtained with primers targeting the mitochondrial *16S rRNA* of different *Demodex* spp. showed a low identity with sequences from other reported species. Therefore, it is possible to assume that the detected *Demodex* species could be specific for *C. prehensilis*, and potentially a new species. Since it is morphologically similar to other *Demodex* species in rodents, but no reference sequences are reported in GenBank from those species, we cannot exclude nor confirm the relationship and phylogeny. This is the first report of a *Demodex* sp. infection in porcupines and the second report of mites in *Coendou* spp. (Gonzalez-Astudillo et al., 2018). Initially it was decided to treat all five *Demodex*-infected animals with moxidectin (Cydectin® 0.1%) at 0.4 mg/kg and 0.8 mg/kg. This treatment was partially effective, as two animals showed negative scrapings, but three family-related animals remained positive. A combination of moxidectin and imidacloprid applied long-term could cure infection with *Demodex musculi* in laboratory mice (Nashat et al., 2018). The dosage and effectiveness of moxidectin in our case might not have been optimal. Nevertheless, the dosage applied did not show any adverse effects. Oral fluralaner was safely applied in a hamster to reduce and eliminate *Demodex* spp. (Brosseau, 2020), and a combination of selamectin and sarolaner applied topically was successful in controlling demodicosis in small rodents (Beck et al., 2021). Based on these reports, we decided to change the treatment to oral sarolaner (available in Switzerland as Simparica® 10 mg tablets). It was applied as one tablet per animal once a month, three times. This treatment was also not completely effective since non-motile *Demodex* sp. were observed in two animals and the skin lesions improved only slightly. Due to the nose skin reactions observed, the treatment with sarolaner was suspended. We attributed this adverse effect to an allergic reaction to any of the components of the palatable tablet, which is optimized for carnivores. One female gave birth to a healthy animal, despite having received all treatments (moxidectin and sarolaner), suggesting that these drugs are likely safe in this species. Since the animals that remained *Demodex*-positive after treatments were all blood-related, increased individual susceptibility and poor immunological response could explain the persistence of infection, similarly to canine demodicosis (Singh and Dimri, 2014). It would be advisable to avoid further reproduction of these animals to reduce the potential susceptibility to demodicosis in the colony. Application of topical treatments was not attempted in the present study to avoid possible injuries of the animal keepers.

As final remarks, this is the first report of demodicosis in coendous, the applied treatment schemes were only partially effective, and there is an apparent individual susceptibility to develop persistent infection.

## Disclaimer and conflicts of interests

All the treatments were performed “off-label” since there is no knowledge of the pharmacokinetics and pharmacodynamics of any medicament in *Coendou prehensilis*. The treatment application was agreed, and all the participants were aware about the potential risks.

We declare that no AI or AI-assisted technologies were used in the writing process.

All the authors are free from conflict of interests which could potentially bias the present study.

## Funding

The present study was supported by internal founding from Papiliorama Foundation and the Institute of Parasitology, 10.13039/100009068University of Bern.

## CRediT authorship contribution statement

**Gastón Moré:** Writing – review & editing, Writing – original draft, Methodology, Formal analysis, Conceptualization. **Peggy Rüegg-van den Broek:** Writing – original draft, Methodology, Funding acquisition, Conceptualization. **Olivier J. Glardon:** Writing – review & editing, Methodology. **Diana S. Gliga:** Writing – review & editing, Methodology. **Caroline F. Frey:** Writing – review & editing, Supervision, Funding acquisition. **Walter Basso:** Writing – review & editing, Supervision, Formal analysis, Conceptualization.

## Declaration of competing interest

All the authors are free from conflict of interests which could potentially bias the present study.

We declare that no AI or AI-assisted technologies were used in the writing process.
